# Effect of different glucogenic to lipogenic nutrient ratios on rumen fermentation and bacterial community *in vitro*

**DOI:** 10.1111/jam.14873

**Published:** 2020-11-21

**Authors:** D. Hua, Y. Zhao, X. Nan, F. Xue, Y. Wang, L. Jiang, B. Xiong

**Affiliations:** ^1^ State Key Laboratory of Animal Nutrition Institute of Animal Sciences Chinese Academy of Agricultural Sciences Beijing China; ^2^ Animal Nutrition Group Department of Animal Sciences Wageningen University & Research Wageningen The Netherlands; ^3^ Beijing Key Laboratory for Dairy Cattle Nutrition Beijing Agricultural College Beijing China

**Keywords:** fermentation, gas production, glucogenic/lipogenic nutrients, *in vitro*, ruminal bacteria

## Abstract

**Aims:**

This study was to investigate the effect of different ratios of glucogenic to lipogenic nutrients on rumen fermentation and the corresponding ruminal bacterial communities.

**Methods and Results:**

Four diets, including glucogenic diet (G), lipogenic diet (L), two mixed diets: GL1 (G: L = 2 : 1) and GL2 (G:L = 1 : 2), served as substrates and were incubated with rumen fluid *in vitro*. The results revealed that the gas production, dry matter digestibility and propionate proportion were significantly increased by the G diet than others. The G diet increased the bacterial genera of *Succinivibrionaceae_UCG_002*, *Succinivibrio*, *Selenomonas_1* and *Ruminobacter* but decreased some cellulolytic bacteria including the *Eubacterium* and several genera in family Ruminococcaceae than others.

**Conclusions:**

When the glucogenic nutrient was above 1/3 of the dietary energy source among the four diets, the *in vitro* incubation had a higher feed digestibility and lower acetate to propionate ratio. Bacterial genera, including *Selenomonas*, *Succinivibrio*, *Ruminobacter*, certain genera in Ruminococcaceae, *Christensenellaceae_R‐7_group* and *Eubacterium*, were more sensitive to the glucogenic to lipogenic nutrients ratio.

**Significance and Impact of the Study:**

The present study provides a new perspective about the effect of dietary glucogenic to lipogenic ingredient ratios on rumen metabolism by comparing end‐products, gas production and bacterial composition via an *in vitro* technique.

## Introduction

Carbohydrate is the dominating nutrition source for ruminants, providing the major energy source for the host animal metabolism and rumen microbial growth (Zhao *et al*. 2016). It has been reported that diets with high lipogenic nutrients, such as forages, CaLCFA (Ca salts of long‐chain fatty acids), tallow or prilled fat, are expected to increase the plasma β‐hydroxybutyrate and the partitioning of metabolic energy into milk and consequently decrease the partitioning of metabolic energy into body reserves (Knegsel *et al*. 2005, 2013). In contrast, glucogenic nutrients, such as grain, non‐fibre carbohydrates, concentrates, starch, glucose infusion and propylene glycol, are expected to decrease the plasma non‐esterified fatty acid level, elevate plasma insulin (Miyoshi *et al*. 2001) and reduce milk fat concentration indicating that glucogenic nutrients stimulate body fat deposition and the partitioning of metabolic energy into body tissue (Ruppert *et al*. 2003). For the ruminants, glucogenic nutrients are originated either from rumen fermentable starch that promotes the production of propionate which is an intermediary precursor for gluconeogenesis or from starch escaping from rumen degradation which is then absorbed as glucose in the small intestine. Lipogenic nutrients stimulate the ruminal production of acetate and butyrate (Knegsel *et al*. 2005). These findings indicate that different glucogenic and lipogenic nutrients lead to different ruminal fermentation products. Another study demonstrated that the complete mix of glucogenic and lipogenic contents made it impossible to ascribe changes in the fermentation products to the concentration changes of specific carbohydrate fractions (Armentano and Pereira 1997). Thus, the confounding effects of different glucogenic to lipogenic nutrient ratios on the rumen fermentation products are still not clear.

The *in vitro* technique which is more convenient and time‐saving than the *in vivo* is widely used to estimate the feed digestibility using the dry matter digestibility (DMD; Tilley and Terry 1963) and gas production (Menke and Steingass 1988), respectively. Ruminal microbiota plays a key role in the feed digestion and the production of gas, volatile fatty acid (VFA) and ammonia‐nitrogen (NH_3_‐N) in the rumen (Patra and Yu 2014). Ruminants hold a large variety of micro‐organisms in their rumen including bacteria, protozoa, fungi and archaea (Kim *et al*. 2011). Although they are the smallest in size, bacteria account for approximately 50% of total microbial volume and are the most investigated population (Fernando *et al*. 2010). In accordance with their main metabolic activity, rumen bacteria are classified into different groups, including amylolytic (e.g. *Selenomonas ruminantium*, *Streptococcus bovis*), fibrolytic (e.g. *Fibrobacter succinogenes*, *Ruminococcus flavefaciens* and *Ruminococcus albus*), proteolytic (e.g. *Prevotella* spp.), lipolytic (e.g. *Anaerovibrio lipolytica*), lactate producers (e.g. *S. bovis* and *S. ruminantium*) and lactate consumers (e.g. *Megasphaera elsdenii*; Belanche *et al*. 2012). In addition, it was also reported that the bacterial functions were influenced by multiple factors including the type of feed, rumen environment and interaction with other bacteria (Sawanon and Kobayashi 2006). Some non‐fibrolytic bacteria, such as *Treponema bryantii* (Kudo *et al*. 1987), *Prevotella ruminicola* (Fondevila and Dehority 1996) and *S. ruminantium* (Koike *et al*. 2003), can activate fibrolytic bacteria through an interaction termed ‘cross‐feeding’. This interaction proved that both fibrolytic bacteria and non‐fibrolytic bacteria are important for fibre degradation in the rumen (Wolin *et al*. 1997). Based on these previous studies, the fermentation end‐products under different ratios of glucogenic to lipogenic nutrients might be attributed to the changes of bacteria as well as the interaction between bacteria. Thus, the comprehensive characterization of bacterial community is essential to understand the effects of glucogenic to lipogenic nutrient ratios on the rumen fermentation end‐products.

Therefore, we hypothesized that different ratios of glucogenic to lipogenic ingredients might impact the rumen bacteria composition, thereby resulting in different fermentation products. To test this hypothesis, the present study, by integrating Illumina sequencing of 16S rRNA gene amplicons, investigated the changes of rumen bacterial community and their fermentation profiles in response to various ratios of glucogenic to lipogenic ingredients via an *in vitro* model.

## Materials and Methods

Animal care and procedures were operated following the Chinese guidelines for animal welfare and approved by the Animal Care and Use Committee of the Chinese Academy of Agricultural Sciences (approval number: IAS2019‐6). Six rumen‐cannulated Holstein dairy cows served as ruminal fluid donors for all three trial runs. The cows were fed a total mixed ration containing (DM basis) 45% concentrate, 20% grass hay, and 35% corn silage, three times daily and had free access to water.

The experimental diets were designed as follows: the glucogenic diet (G) using corn and corn silage as main energy sources; the lipogenic diet (L) using sugar beet pulp and alfalfa silage as main energy sources; the mixed diet one (GL1): 2/3 of the energy sources were from corn and corn silage and 1/3 were from sugar beet pulp and alfalfa silage; the mixed diet two (GL2): 1/3 of the energy sources were from corn and corn silage and 2/3 were from sugar beet pulp and alfalfa silage. Besides, the soybean meal, oat and alfalfa hay, and calcium hydrogen phosphate were used to balance the nutritional requirement. All diets were on an isocaloric basis and their composition and chemical analysis are shown in Table 1.

**Table 1:** Composition and nutrient levels of experimental diets

Items	G	GL1	GL2	L
Ingredient (% DM)				
Corn	28·0	20·0	10·0	
Sugar beet pulp		12·6	20·8	28·0
Soybean meal	18·5	16·8	14·6	12·0
Oat hay	5·0	7·1	14·2	19·0
Alfalfa hay	10·0	10·0	10·0	10·0
Corn silage	38·0	23·5	10·0	
Alfalfa silage		10·0	19·0	30·0
Calcium hydrogen phosphate	0·5		1·4	1·0
Composition (g kg^−1^ DM)*				
CP	174·4	177·7	175·4	174·6
EE	24·3	22·3	20·6	20·4
Starch	280·0	207·6	121·0	41·1
NDF	326·0	402·8	482·5	562·2
ADF	197·9	243·9	294·1	348·9
NE_L_ MJ kg^−1^ DM	7·3	7·7	7·6	7·9

G, glucogenic diet; GL1, glucogenic ingredient: lipogenic ingredient = 2: 1; GL2, glucogenic ingredient: lipogenic ingredient = 1: 2; L, lipogenic diet.

CP = crude protein; EE = ether extract; NDF = neutral detergent fibre; ADF = acid detergent fibre; NE_L_ = net energy for lactation and calculated according to NRC (2001).

* Nutrient composition of the experimental diets was calculated according to NRC (2001).

### *In vitro* incubation

A ground dry matter (1·0 mm) of each diet was used as the substrate in the incubation. Fresh ruminal fluid from two cows (two different cows for each run) was collected through rumen fistula separately 1 h after morning feeding, combined in equal portions and strained through four layers of cheesecloth. The inoculation and incubation procedures were operated as described by Shen *et al*. (2017). Briefly, 0·5 g substrate was preloaded into a 150 ml serum vial. The buffered medium was prepared anaerobically at 39°C according to Menke and Steingass (1988). The anaerobic buffer medium (50 ml per vial) and rumen fluid inoculum (25 ml per vial) were added into the vials successively. All the inoculating procedures were conducted in a water bath of 39°C under a stream of CO_2_. Each serum vial was sealed with a butyl rubber stopper and secured with an aluminium seal. Three replicate vials were prepared for each diet treatment in each run. All the incubation vials were individually connected to the gas inlet of an automated gas production recording system (AGRS, Fig. S1a,b) and then incubated under 39°C for 48 h, as described by Zhang and Yang (2011). The *in vitro* incubation was repeated for triple runs with different cows as ruminal fluid donors.

### Sample collection and processing

After 48 h of incubation, the total gas produced by fermentation in each vial was recorded by the AGRS. All vials were withdrawn from the incubator and transferred into an ice–water mixture to terminate the incubation. The pH of the whole contents was measured using a portable pH–meter (PHB‐4, INESA, Shanghai, China). Then, the fermented substrates were filtered through a nylon bag (50 *µ*m of the pore size, weighed after drying at 65°C for 48 h before use). The bag together with filtered residue was washed under running water until the effluent was clear and then dried at 65°C for 48 h. Bags and contents were weighed to estimate the DMD. 1 ml of supernatant was preserved by adding 0·2 ml of 25% metaphosphoric acid for VFA measurement by gas chromatography (7890B, Agilent Technologies) according to the method described by Mao *et al*. (2008). Another 1 ml of supernatant was used to determine the NH_3_–N concentration by the phenol–hypochlorite method (Shen *et al*. 2017). Finally, five supernatant samples per diet of all three runs were randomly chosen to do DNA extractions and subsequent microbial analysis.

### DNA extraction

Microbial DNA was extracted from 5 ml supernatant using the QIAamp DNA Stool Mini Kit (Qiagen, Hilden, Germany) according to the manufacturer's instructions with the addition of a bead‐beating step as described in a previous study (Pan *et al*. 2017). Briefly, the supernatant sample was homogenized with 0·5 g zirconium beads (0·5 mm diameter) and 800 ml CTAB buffer using a Mixer Mill MM 400 (Retsch, Haan, Germany) with the vibrational frequency of 180 ɡ and grinding time of 60 s. Then the mixture was incubated at 70°C for 20 min to increase DNA yield. The supernatant was further processed using QIAamp kits according to the manufacturer’s instructions. The integrity and length of the extracted DNA were assessed by agarose gel (1%) electrophoresis on gels containing 0·5 mg ml^−1^ ethidium bromide and quantified using a NanoDrop spectrophotometer ND–1000 (Thermo Scientific, Waltham, MA). DNA was stored at −80°C until analysis.

### Sequencing data processing and analysis

The V3–V4 hypervariable regions of the bacterial 16S rRNA gene were amplified with primers 338F (5′–ACTCCTACGGGAGGCAGCAG–3′) and 806R (5'–GGACTACHVGGGTWTCTAAT–3') by thermocycler PCR system (GeneAmp 9700, ABI, Vernon, CA) (Ye *et al*. 2016; Pan *et al*. 2017), where the barcode was an eight–base sequence unique to each sample. PCRs were performed in triplicate 20 *μ*l mixture containing 4 *μ*l of 5 × FastPfu Buffer, 2 *μ*l of 2·5 mmol dNTPs, 0·8 *μ*l of each primer (5 *μ*mol), 0·4 *μ*l of FastPfu Polymerase and 10 ng of rumen microbial DNA. PCR amplification started with a 3 min of pre‐denaturation at 95°C, followed by 27 cycles of denaturation (95°C for 30 s), annealing (55°C for the 30 s) and elongation (72°C for 45 s) steps, and a final extension at 72 °C for 10 min. The PCR amplicons were extracted from 2% agarose gels and further purified using the AxyPrep DNA Gel Extraction Kit (Axygen Biosciences, Union City, CA) and quantified using QuantiFluor™–ST (Promega, Madison, WI) according to the manufacturer’s protocol. Purified amplicons were pooled in equimolar and paired‐end sequenced (2 × 300) on an Illumina MiSeq platform (Illumina, San Diego) according to the standard protocols by Majorbio Bio‐Pharm Technology Co. Ltd. (Shanghai, China) (Jin *et al*. 2017).

Raw fastq files were quality filtered using Trimmomatic (Bolger *et al*. 2014), and merged using FLASH (Magoc and Salzberg 2011), based on the following criteria: (i) the reads were truncated at any site receiving an average quality score of <20 over a 50 bp sliding window; (ii) sequences of each sample were separated according to barcodes (exactly matching); primers (allowing two nucleotide mismatching) and reads containing ambiguous bases were removed; (iii) only sequences whose overlaps were longer than 10 bp were merged according to their overlap with mismatch no more than 2 bp. Operational taxonomic units (OTUs) were clustered with a cut‐off of 0·03 (97% similarity) using UPARSE (Edgar *et al*. 2011) with a novel greedy algorithm that performs chimaera filtering and OTU clustering simultaneously. The taxonomy of each 16S rRNA gene sequence was aligned with the RDP Classifier algorithm and compared with the Silva (SSU123) 16S rRNA database (Pruesse *et al*. 2007) with a confidence threshold of 70% (Amato *et al*. 2013). Alpha diversity was estimated with the normalized reads using the based coverage estimator Shannon, Simpson, ACE, Chao1 and Coverage indices. The principal coordinate analysis (PCoA) was performed based on the Bray–Curtis dissimilarity (Mitter *et al*. 2017), and the significant differences between samples were tested by an analysis of similarity (ANOSIM) in QIIME with 999 permutations (R Core Team 2013). Tabular representation of the relative abundance of microbial diversity at phylum and genus levels was counted depending on the taxonomic data.

In addition to bacterial community structure analysis, the method of Phylogenetic Investigation of Communities by Reconstruction of Unobserved States (PICRUSt) was also used to predict the metagenomic potential functions of ruminal bacteria based on 16S rRNA data. First, the closed OTU table was performed using the sampled reads against the Greengenes database (13.5) with QIIME (Liu *et al*. 2016). Next, the table was normalized by 16S rRNA copy number. Then, the metagenome functions were predicted and the data were exported into the Kyoto Encyclopedia of Genes and Genomes (KEGG) pathways using PICRUSt (Langille *et al*. 2013). The difference of the predicted functions among diets was determined by one‐way analysis of variance with SAS 9.3 (SAS Institute Inc., Cary, NC).

### Statistical analysis

Data were checked for normal distribution and homogeneity by Shapiro–Wilk’s and Levene’s tests by SAS 9.3 (SAS Institute Inc.). Rumen fermentation parameters, alpha diversity index and bacterial relative abundance were analysed using PROC MIXED by SAS 9.3 (SAS Institute Inc.) with the following model:

$$Y_{\mathrm{ij}}=\mu +D_{i}+R_{j}+e_{\mathrm{ij}},$$

where *Y_ij_* is the dependent variable, *µ* the overall mean, *D_i_* the fixed effect of diet (*i* = 1–4), *R_j_* is the random effect of run (*j* = 13) and e_ij_ is the random residual error. Significance was declared at *P ≤ *0·05 and a tendency was considered at 0·05 < *P* *≤* 0·10. Pearson correlation coefficients between the relative abundance of bacterial genera (the top 20 genera) and the ruminal fermentation variables were calculated using sas 9.3 (SAS Institute Inc., Cary, NC). A significant correlation was considered at *P ≤ *0·05.

## Results

### Effect of glucogenic to lipogenic nutrient ratios on rumen fermentation parameters

The fermentation characters are shown in Table 2. As lipogenic ingredients increased, gas production had a significantly decreasing trend (*P *<* *0·05), and the DMD showed a similar trend (*P <* 0·05). The pH of the G and GL1 diets was significantly lower than that of the diet GL2 and L (*P <* 0·05). The NH_3_–N concentration of the G diet was significantly higher than that of the GL2 and L diets (*P* < 0·05). For VFA contents, the L diet significantly increased the proportion of acetate than the other three diets (*P <* 0·05), while the diet G significantly increased the propionate proportion than others (*P < *0·05). Consequently, the acetate to propionate ratio in the diet G was the lowest and was the highest in the diet L (*P < *0·05).

**Table 2:** Effects of glucogenic to lipogenic nutrient ratios on rumen fermentation parameters

Item*	G		GL1	GL2	L	SEM	*P* value
Gas production (ml g^−1^ DM)	135·43^a^		116·10^b^	106·73^c^	92·24^d^	2·885	<0·001
DMD (%)	87·64^a^		83·22^b^	81·39^b^	75·82^c^	0·823	<0·001
pH	6·60^b^		6·61^b^	6·68^a^	6·72^a^	0·011	<0·001
NH_3_–N (mmol l^−1^)	38·97^a^		33·64^a,b^	31·77^b^	29·34^b^	1·218	<0·001
tVFA (mmol l^−1^)	129·29		129·36	128·03	119·28	1·569	0·100
VFA contents (% of tVFA)							
Acetate		55·64^c^	57·93^b^	58·93^b^	60·25^a^	0·309	<0·001
Propionate		23·62^a^	21·33^b^	21·32^b^	20·47^b^	0·261	<0·001
A/P		2·36^c^	2·74^b^	2·76^b^	2·94^a^	0·044	<0·001
Isobutyrate		4·60	4·47	4·36	4·45	0·059	0·580
Butyrate		9·74	9·80	9·57	9·61	0·102	0·687
Isovalerate		5·84	5·93	5·67	5·59	0·069	0·304
Valerate		0·56	0·55	0·51	0·50	0·020	0·658

G, glucogenic diet; GL1, glucogenic ingredient: lipogenic ingredient = 2: 1; GL2, glucogenic ingredient: lipogenic ingredient = 1: 2; L, lipogenic diet.

DMD, dry matter digestibility; tVFA, total volatile fatty acid; A/P = acetate/propionate; SEM = standard error of the mean.

^a,b,c^means values with different letters differed significantly within a row (*P* < 0·05).

### Effect of glucogenic to lipogenic nutrient ratios on rumen bacterial communities

**Figure 1:**
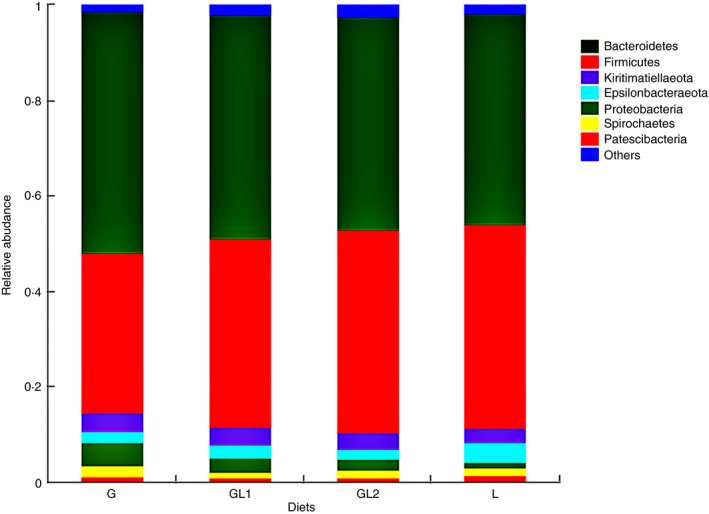
Composition of the top predominant bacteria phyla in the rumen fluid. Only the phyla with the relative abundance above 0·01 in at least one sample were shown in the figure. G, glucogenic diet; GL1, glucogenic ingredient: lipogenic ingredient = 2: 1; GL2, glucogenic ingredient: lipogenic ingredient = 1: 2; L, lipogenic diet. *Bacteroidetes*, *Firmicutes* and *Kiritimatiellaeota* were the three dominant phyla, representing 46·8%, 39·1% and 3·6% of the total sequences. [Colour figure can be viewed at wileyonlinelibrary.com]

Across all samples, 1 064 890 qualified sequence reads were acquired with an average read length of 418 bases, all reads were assigned to 2089 OTUs using a cut‐off of 97% sequence similarity. The total number of reads from each sample varied from 28 702 to 49 765 with an average of 36 951. Among the bacterial community, 20 phyla were identified across all samples (Table S1). The predominant phyla with relative abundance above 1% in at least one sample are shown in Fig. 1. *Bacteroidetes*, *Firmicutes* and *Kiritimatiellaeota* were the three dominant phyla, representing 46·8, 39·1 and 3·6% of the total sequences on average, respectively. *Proteobacteria*, *Epsilonbacteraeota*, *Spirochaetes* and *Patescibacteria* represented an average of 2·9, 2·8, 1·7 and 1·1%, separately, of the total sequences. The other phyla, such as *Armatimonadetes*, *Planctomycetes* and *Verrucomicrobia*, were not consistently present in all ruminal samples (Table S1).

As for the alpha diversity estimates (Table 3), the G diet significantly decreased the number of OTUs compared with GL2 and L diets. The ACE and Chao estimates of richness in the GL2 diet were significantly higher than that of the G diet.

**Table 3:** Effects of glucogenic to lipogenic nutrient ratios on the alpha diversity

Estimators	G	GL1	GL2	L	SEM	*P* value
OTU	1467^b^	1527^ab^	1586^a^	1553^a^	13·960	0·006
ACE	1760^b^	1803^ab^	1850^a^	1817^ab^	12·328	0·005
Chao1	1797^b^	1831^ab^	1866^a^	1848^ab^	11·108	0·013
Shannon	5·745	5·787	5·852	5·777	0·032	0·694
Simpson	0·015	0·010	0·010	0·013	0·001	0·419
Coverage	0·990	0·991	0·991	0·991	0·0003	0·172

G, glucogenic diet; GL1, glucogenic ingredient: lipogenic ingredient = 2: 1; GL2, glucogenic ingredient: lipogenic ingredient = 1: 2; L, lipogenic diet; SEM = standard error of the mean.

^a,b^means values with different letters differed significantly within a row (*P* < 0·05).

**Figure 2:**
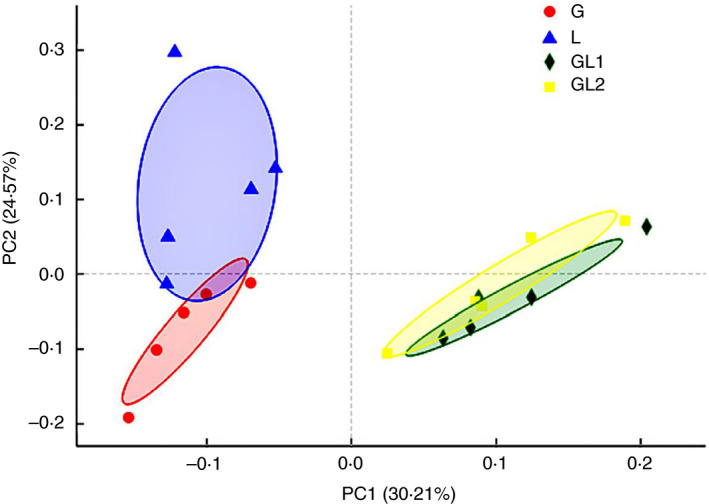
Principal coordinate analysis (PCoA) of bacteria community structures in rumen fluid. G, in the red circle, glucogenic diet; GL1, in the green diamond, glucogenic ingredient: lipogenic ingredient = 2: 1; GL2, in the yellow square, glucogenic ingredient: lipogenic ingredient = 1: 2; L, in the blue triangle, lipogenic diet. The diet G was separated from the diet L along PC2, which explained 24·6% of the total variation. The diets GL1 and GL2 were clearly separated from the diets G and L along PC1, which explained 30·2% of the total variation. [Colour figure can be viewed at wileyonlinelibrary.com]

The PCoA result is shown in Fig. 2. The diet GL1 and GL2 were clearly separated from the diet G and L along PC1, which explained 30·2% of the total variation, while G was separated from the diet L along PC2, which explained 24·6% of the total variation. The separation between GL1 and GL2 was not significant.

At the phylum level, the top five phyla which were influenced or potentially influenced by the treatments are listed in Table 4. The G diet significantly increased the relative abundance of *Bacteroidetes* and *Proteobacteria*, while the L diet significantly increased the relative abundance of *Firmicutes* and *Patescibacteria* (*P < *0·05).

**Table 4:** Effect of glucogenic to lipogenic nutrient ratios on the relative abundances of bacterial phyla and genera in rumen fluid (%)

Phyla	Family	Genus/other	Diets				SEM	*P* value
			G	GL1	GL2	L		
*Bacteroidetes*		Total	50·881^a^	47·370^ab^	45·051^b^	44·480^b^	0·9478	0·0891
	Rikenellaceae unclassified	*SP3‐e08*	0·114^b^	0·066^c^	0·077^bc^	0·170^a^	0·0110	0·0002
		*Unclassified_o__Bacteroidales*	0·049^b^	0·092^a^	0·099^a^	0·101^a^	0·0068	0·0069
*Firmicutes*		Total	33·191^b^	39·190^ab^	42·121^a^	42·270^a^	1·2719	0·0394
	Ruminococcaceae	*Ruminococcaceae_UCG_group*	3·699^b^	2·809^c^	2·980^bc^	4·629^a^	0·2356	0·0005
		*Ruminococcaceae_NK4A214_group*	2·331^b^	4·297^a^	4·141^a^	2·733^b^	0·2305	<0·0001
		*Ruminococcus_2*	0·964^b^	1·373^a^	1·055^b^	0·547^c^	0·0783	<0·0001
		*Ruminococcus_1*	0·174^c^	0·265^bc^	0·410^a^	0·354^ab^	0·0300	0·0156
		*[Ruminococcus]_gauvreauii_group*	0·331^b^	1·043^a^	1·063^a^	0·417^b^	0·0846	<0·0001
		*Saccharofermentans*	0·420^b^	0·289^c^	0·346^bc^	0·563^a^	0·0299	0·0011
		*Unclassified_f__Ruminococcaceae*	0·236^c^	0·268^bc^	0·337^ab^	0·366^a^	0·0168	0·0092
	Lachnospiraceae	*Lachnospiraceae__group*	1·661^b^	1·654^b^	1·806^b^	3·123^a^	0·2117	0·0068
		*unclassified_f__Lachnospiraceae*	0·381^b^	0·543^ab^	0·622^a^	0·714^a^	0·0399	0·0104
		*Oribacterium*	0·546^b^	0·609^b^	0·859^b^	1·509^a^	0·1260	0·0128
		*Eubacterium*	1·343^c^	2·498^b^	3·024^a^	2·006^b^	0·1715	0·0001
		*Acetitomaculum*	0·184^b^	0·776^a^	0·839^a^	0·213^b^	0·0744	<0·0001
	Christensenellaceae	*Christensenellaceae_R‐7_group*	1·241^b^	4·992^a^	4·647^a^	1·617^b^	0·4523	<0·0001
	Family_XIII	*Family_XIII_AD3011_group*	0·773^b^	2·006^a^	2·359^a^	1·291^b^	0·1643	<0·0001
		*Anaerovorax*	0·472^b^	0·310^c^	0·348^bc^	0·641^a^	0·0381	0·0016
	Veillonellaceae	*Selenomonas_1*	0·554^a^	0·285^b^	0·271^b^	0·263^b^	0·0354	0·0011
*Patescibacteria*		Total	1·061^b^	0·921^b^	0·930^b^	1·460^a^	0·0665	0·0067
	Saccharimonadaceae	*Candidatus_Saccharimonas*	0·885^b^	0·664^b^	0·650^b^	1·162^a^	0·0598	0·0009
*Proteobacteria*		Total	4·720^a^	2·951^b^	2·311^b^	1·321^c^	0·3329	<0·0001
	Succinivibrionaceae	*Ruminobacter*	1·653^a^	0·791^b^	0·592^b^	0·161^c^	0·1289	<0·0001
		*Succinivibrionaceae_UCG_002*	1·292^a^	0·316^b^	0·158^b^	0·077^b^	0·1342	0·0002
		*Succinivibrio*	0·523^a^	0·283^b^	0·152^c^	0·128^c^	0·0394	<0·0001
		*Pseudomonas*	0·073^b^	0·481^a^	0·362^a^	0·003^b^	0·0512	<0·0001
*Actinobacteria*		Total	0·130^b^	0·611^a^	0·690^a^	0·140^b^	0·0729	<0·0001
	Eggerthellaceae	*DNF00809*	0·023^b^	0·171^a^	0·192^a^	0·027^b^	0·0213	0·0001
	Atopobiaceae	*Atopobium*	0·041^b^	0·131^a^	0·176^a^	0·039^b^	0·0174	0·0016

G, glucogenic diet; GL1, glucogenic ingredient: lipogenic ingredient = 2: 1; GL2, glucogenic ingredient: lipogenic ingredient = 1: 2; L, lipogenic diet.

^a,b,c^ means values with different letters differed significantly within a row (*P* < 0·05); SEM = standard error of the mean. Only the top 25 of influenced genera with a relative abundance of ≥0·1% in at least one sample were listed.

At the genus level, a total of 260 bacteria genera were identified. The top 25 of the influenced genera (*P* < 0·05) with a relative abundance of ≥0·1% in at least one sample are listed in Table 4. Specifically, the L diet significantly increased the proportions of seven genera compared to others (*P *< 0·05), including *Ruminococcaceae_UCG_group*, *Lachnospiraceae_group*, *Oribacterium*, *Anaerovorax*, *Saccharofermentans*, *SP3–e08* and *Candidatus_Saccharimonas* while significantly decreased the relative abundance of *Ruminococcus_2* and *Ruminobacter*. Compared to the GL1, GL2 and L diets, four genera were increased by the G diet (*P *< 0·05), including *Selenomonas_1*, *Ruminobacter*, *Succinivibrionaceae_UCG_002* and *Succinivibrio*. Besides, compared to the diet G and L, the GL1 and GL2 diets increased the relative abundance of *Ruminococcaceae_NK4A214_group*, *[Ruminococcus]_gauvreauii_group*, *Christensenellaceae_R–7_group*, *Acetitonmaculum*, *unclassified_o_Bacteroidales*, *Pseudomonas*, *DNF00809*, *Family_XIII_AD3011_group* and *Atopobium* (*P *< 0·05).

### Correlation analysis between the relative abundance of bacterial genera and the fermentation parameters

**Figure 3:**
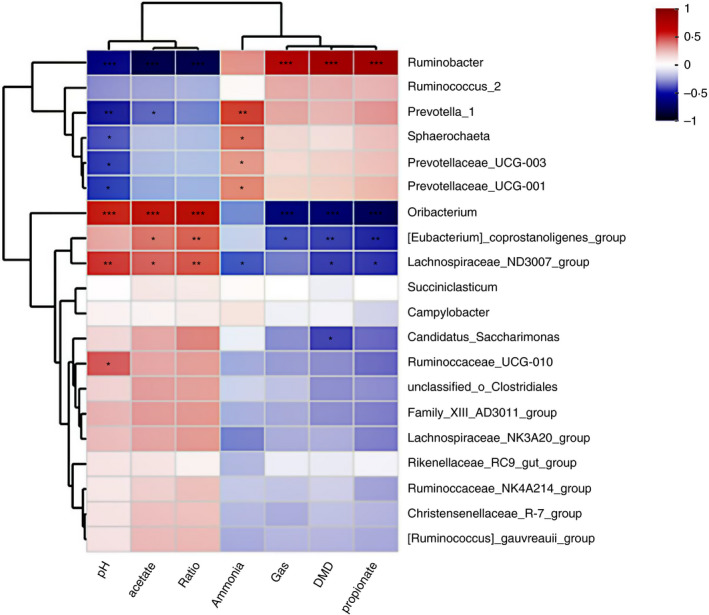
Correlation analysis between the relative abundance of the top 20 bacterial genera and influenced ruminal fermentation parameters including pH, acetate proportion, acetate/propionate ratio (ration), ammonia‐nitrogen (ammonia), gas volume, dry matter digestibility (DMD) and propionate proportion. The red represents a positive correlation, the blue represents a negative correlation. * means the correlation is in a significant level (*P* < 0·05), **means the correlation is in extremely significant level (*P* < 0·01). The genera *Rumiinobacter, Lachnospiraceae_ND3007_group*, *Eubacterium_coprostanoligenes_group* and *Oribacterium* were significantly correlated with most variables. [Colour figure can be viewed at wileyonlinelibrary.com]

As shown in Fig. 3, the genus of *Ruminobacter* was positively correlated with the gas production, DMD, and propionate proportion, but negatively correlated with the pH, acetate proportion and acetate to propionate ratio. The genera of *Prevotella_1*, *Sphaerochaeta*, *Prevotellaceae_UCG_003* and *Prevotellaceae_UCG_001* were negatively correlated with the pH but positively correlated with the concentrations of NH_3_–N. The *prevotella_1* was negatively correlated with the acetate proportion. The *Oribacterium* was positively correlated with the pH, acetate proportion and acetate to propionate ratio, but negatively correlated with the gas production, DMD and propionate proportion. The *[Eubacterium]_coprostanoligenes_group* was positively correlated with the acetate proportion and acetate to propionate ratio but negatively correlated with the gas production, DMD and propionate proportion. The *Lachnospiraceae_ND3007_group* was positively correlated with the pH, acetate proportion and acetate to propionate ratio but negatively correlated with the NH_3_–N concentration, DMD and propionate proportion. The *Candidatus_Saccharimonas* was negatively correlated with the DMD, whereas the *Ruminococcaceae_UCG_010* was positively correlated with the pH.

### Functional analysis

**Figure 4:**
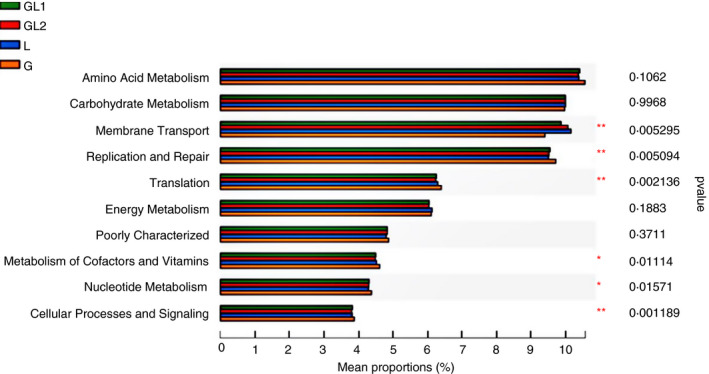
Effect of different glucogenic to lipogenic nutrient ratios on the relative abundance of KEGG pathways of the rumen bacteria. Only the top 10 relative abundance of the inferred functions were presented. *means the difference is in a significant level (*P* < 0·05), **means the correlation is in extremely significant level (*P* < 0·01). G, glucogenic diet; GL1, glucogenic ingredient: lipogenic ingredient = 2: 1; GL2, glucogenic ingredient: lipogenic ingredient = 1: 2; L, lipogenic diet. KEGG = Kyoto Encyclopedia of Genes and Genomes. Amino acid metabolism, carbohydrate metabolism, membrane transport, and replication and repair were the most abundant functions in all samples. There were six KEGG categories disturbed by diets. [Colour figure can be viewed at wileyonlinelibrary.com]

To characterize the functional alterations of ruminal bacteria among different diets, the functional composition profiles were predicted from 16S rRNA sequencing data with PICRUSt (Table S2). The top 10 KEGG pathways of level 2 are illustrated in Fig. 4. Amino acid metabolism, carbohydrate metabolism, membrane transport, and replication and repair were the most abundant functions in all samples. Multiple KEGG categories were disturbed by diets. Compared with other diets, the diet G had a significantly higher relative abundance of translation, metabolism of cofactors and vitamins, and cellular processes and signalling, but had a lower relative abundance of membrane transport (*P* < 0·05). Compared to the diet GL2 and L, the G diet could significantly increase the relative abundance of replication and repair as well as nucleotide metabolism (*P* < 0·05).

## Discussion

### Effects of glucogenic to lipogenic nutrient ratios on the major bacterial community involved in feed digestion

Rate and extent of starch digestion in the rumen were determined by several factors, including the source of dietary starch, diet composition, grain processing and degree of adaptation of ruminal microbiota to the diet (Huntington 1997). The rumen amylolytic bacteria convert starch to glucose, which is then used for growth and provides energy for the synthesis of microbial proteins. Reported amylolytic bacteria included *S. bovis*, *Bacteroides amylophilus*, *Prevotella spp*., *Succinimonas amylolytica*, *S. ruminantium* and *Butyrivibrio* spp. (Giraud *et al*. 1994; Huntington 1997), some of whose amylolytic activities have been demonstrated *in vitro*, previously (Minato and Suto 1979; Miura *et al*. 1983; Cotta 1988; Xia *et al*. 2015). Pure culture studies have demonstrated that most of these starch‐degrading bacteria have more energy supply sources not only from starch but also from other nutrients (Kotarski *et al*. 1992; Klieve *et al*. 2007). Thus, their dominant presence in ruminants fed diets with high starch may not be necessarily associated with their starch‐hydrolysing capacity (Klieve *et al*. 2012). This might explain that the dominant amylolytic bacteria did not differ among diets in the present study. However, the relative abundance of *Selenomonas_1*, *Ruminobacter*, *Succinivibrionaceae_UCG_002* and *Succinivibrio* were significantly higher in the G diet than the other three diets. These increased bacteria genera might be recognized as being sensitive to the dietary glucogenic nutrients.

Generally, the apparent digestibility of starch was nearly twice as high as that of neutral detergent fibre (NDF) as described by Firkins *et al*. (2001). The cellulolytic bacteria are known as the dominating contributors for fibre degradation. *Fibrobacter succinogenes*, *Ruminococcus flavefaciens* and *Ruminococcus albus* are recognized as the most active cellulolytic bacteria (Wanapat *et al*. 2014). *Butyrivibrio*, *Oscillibacter*, *Pseudobutyrivibrio* and *Eubacterium* are also classified as cellulolytic bacterial genera (Thoetkiattikul *et al*. 2013). Besides, some unclassified groups, such as the taxa assigned to Lachnospiraceae, Christensenellaceae, Ruminococcaceae, Rikenellaceae, Prevotellaceae and Bacteroidales had been proved tightly attaching to fibre in the rumen, suggesting that they might play a significant role in the ruminal digestion of fibre (Liu *et al*. 2016). In the present study, the GL1, GL2 and L diets compared to the G diet significantly increased the relative abundance of the fibrolytic bacterial genera, including *Ruminococcus_2*, *Ruminococcaceae_UCG_group*, *Ruminococcaceae_NK4A214_group*, *Ruminococcus_gauvreauii_group*, *Ruminococcus_1* (Krause *et al*. 2003), some unclassified taxa *(unclassified_f_Lachnospiraceae*, *unclassified_f_*Ruminococcaceae, *unclassified_o_Bacteroidales*) (Liu *et al*. 2016), and the genus of *[Eubacterium]_group* (Thoetkiattikul *et al*. 2013). In addition, compared to the diet L, the two mixed diets gained a higher number of the *Ruminococcaceae_NK4A214_group*, *Ruminococcus_2*, *Christensenellaceae_R‐7_group* and *Ruminococcus_gauvreauii_group*, but gained a lower number of *Ruminococcaceae_UCG_group* and *Lachnospiraceae_group*. These changes illustrated that when the dietary lipogenic nutrients were higher than 2/3 of the dietary energy source, some bacteria in the genera *Ruminococcaceae_NK4A214_group*, *Ruminococcus_gauvreauii_group*, *Ruminococcus_2* and *Christensenellaceae_R‐7_group* would rapidly decrease, while other bacteria in the genera *Ruminococcaceae_UCG_group* and *Lachnospiraceae_group* would increase.

Furthermore, according to the correlated analysis (Fig. 3), the DMD and gas production were positively correlated with the genus of *Ruminobacter*. The previous study also reported that bacteria related to *Ruminobacter* would dominate in the ruminal ecosystem when cows were introduced to a high grain diet (Klieve *et al*. 2012). The genus *Ruminobacter* might play an important role in leading to the difference in fermentation end‐products.

In summary, these sensitive amylolytic and cellulolytic bacteria might lead to the difference in the feed digestion. In addition, some genera whose functions were not clear were also influenced by the diets, including *SP3–e08*, *Pseudomonas*, *DNF00809* and *Atopobium*. Their functions and contribution to fermentation products still need further research.

### Effects of glucogenic to lipogenic nutrient ratios on VFA and related bacteria

The dietary carbohydrate was finally fermented to VFA by microbes in the rumen. The major ingredients of VFA contain acetate, propionate and butyrate, whose proportions are mainly affected by the NDF to starch ratio in the diet. Ruminants fed a high proportion of dietary starch produced proportionally more propionate than those fed a high forage diet which produced more acetate (Wu *et al*. 1994; Marounek and Bartos 2010; Wang *et al*. 2016). Propionate is produced in the ruminal ecosystem by two major pathways. One is the succinate pathway in which the propionate is produced directly by decarboxylating of succinate (Jeyanathan *et al*. 2014). This pathway involves a large number of microbes, such as fumarate reducers (e.g. *Wolinella succinogenes*), succinate producers (e.g. *Fibrobacter succinogenes*) and succinate utilizers (e.g*. S. ruminantium*) (Jeyanathan *et al*. 2014). Succinate is produced by the members in the genus *Succinivibrio* as their key fermentation end‐product (Pope *et al*. 2011), which is then digested to propionate by the members of *Selenomonas* (e.g. *S. ruminantium*) via the succinate pathway (Scheifinger and Wolin 1973). The other one is the acrylate pathway which starts indirectly from lactate via dehydration to acrylate and turns to propionate via reduction reaction (Puniya *et al*. 2015; Zhao *et al*. 2020). Starch is degraded by *S. bovis* and *Lactobacillus spp*. to lactic acid (Hutton *et al*. 2012) which is then utilized by *M. elsdenii*, the major bacteria involved in the acrylate pathway (Hino *et al*. 1994). Other lactate‐utilizing bacteria such as *S. ruminantium*, *Propionibacterum spp*. (Klieve *et al*. 2003) and some strains of the bacterium *P. ruminicola* also play important roles in the acrylate pathway (Wallnofer and Baldwin 1967). In the present study, the greatly increased relative abundance of *Succinivibrio* members (*Succinivibrionaceae_UCG_002* and *Succinivibrio*), *Selenomonas* member (*Selenomonas_1*) and the *Ruminobacter* in the G diet probably contributed to the increased propionate production via the succinate pathway.

The decreased acetate in the G diet can be explained by the reduction of some gram‐positive fibrolytic bacteria, such as *Ruminococcus* spp., which is recognized as the main acetate‐producing bacteria (Jeyanathan *et al*. 2014). The *Anaerosporobacter* and *Saccharofermentans* are also known for producing acetate as the main end‐products (Ziemer 2014). In addition, some unclassified bacteria, such as unclassified bacteria in Ruminococcaceae, Lachnospiraceae and Christensenellacea*e* were reported to be correlated with acetate concentration (Shen *et al*. 2017). In the present study, the increased populations of *Saccharofermentans*, *Anaerovorax*, *Lachnospiraceae_ND3007_group*, and the unclassified groups in Ruminococcaceae, Lachnospiraceae and Christensenellaceae might have also contributed to the improvement of acetate production in the L diet.

In addition, the genus *Oribacterium* was positively correlated with acetate proportion and negatively correlated with the DMD and propionate proportion. This was a newly classified genus proposed by Carlier *et al*. (2004), which was latterly reported to be identified in the rumen of cows fed forage‐based diets (Kong *et al*. 2010; Zened *et al*. 2013) and capable to degrade pectin from plant cell walls in the rumen environment (Kang *et al*. 2019). This could explain their high population in the diet L. To our knowledge, its function related to acetate production was not reported yet, thus it needs further research.

### Effects of glucogenic to lipogenic nutrient ratios on NH_3_–N and related microbes

The NH_3_–N concentration was consistent with the DMD trend, which was towards a lower NH_3_–N concentration as the lipogenic nutrient ratio increased. This result was in line with the study of Beckman and Weiss (2005). Dietary protein is degraded in the rumen to peptides and amino acid, and eventually deaminated into NH_3_–N or incorporated into microbial protein (Bach *et al*. 2005). When the rumen‐digested protein excesses the requirement of ruminal micro‐organisms, the protein is degraded to NH_3_–N which is then metabolized to urea in the liver, and finally excreted in urine (Tamminga 1996). The NH_3_–N accounts for about 34% of the protein requirement for ruminal micro‐organisms. The NH_3_–N concentration in the rumen depends on the balance between the rate of formation and utilization of NH_3_–N by microbes. Amylolytic bacteria tended to be more proteolytic than fibrolytic bacteria (Siddons and Paradine 1981; Wallace *et al*. 1997; Ferme *et al*. 2004). It was also reported that amylases had positive effects on protein degradation in the rumen (Tománková and Kopečný 1995). In addition, the cellulolytic microbes grow slowly with low maintenance requirements, solely take NH_3_–N as their nitrogen source; while the amylolytic microbial communities grow fast, require more nitrogen for maintenance, and have multiple nitrogen sources including NH_3_–N, peptides and AA (Bach *et al*. 2005). This preferential use of nitrogen sources by ruminal bacteria was in agreement with the difference of NH_3_–N concentrations in the present study. To summarize, the G diet tended to increase protein degradation and decrease the nitrogen utilization by ruminal bacteria, which might partially explain the increased ruminal NH_3_–N concentration.

In addition, some species in the genus *Prevotella* were considered as ammonia‐producing bacteria, such as *Prevotella ruminantium* and *Prevotella bryantii* (Ferme *et al*. 2004). This could probably explain the positive correlationship between the NH_3_–N concentration and the genus *Prevotella*.

### Effects of glucogenic to lipogenic nutrient ratios on metagenomic functions

Diets can reshape the bacterial communities in the rumen; consequently, the functions of ruminal bacteria may be altered along with the changes. A tool of PICRUSt is developed for inferring the functional potential of microbial communities based on 16S data, which needs little extra skill or cost compared to the metagenomics and metatranscriptomics technologies (Wilkinson *et al*. 2018). In the present study, the PICRUSt was carried out to predict the functional alterations of rumen bacteria associated with different ratios of glucogenic to lipogenic ingredients. In the results, the most abundant functional categories contained amino acid metabolism, carbohydrate metabolism, replication and repair, membrane transport and translation, which were proved to be fundamental for the growth and reproduction of bacteria (Seddik *et al*. 2019). The G diet was predicted to lower the pathway of membrane transport than other diets. The membrane transport function is significant for microbes in the communication with the rumen environment, such as capturing nutrients and secreting functional proteins or substances (Konishi *et al*. 2015; Zhang *et al*. 2017a). The relation between bacterial membrane transport function and their digesting capacity in the rumen deserves further research. In addition, several functions, such as translation, cofactors and vitamins metabolism, replication and repair, and cellular processes and signalling, were enriched by diet G compared to other diets. These results were partly in line with the previous report (Zhang *et al*. 2017a; Zhang *et al*. 2017b). These improved functions in diet G might relate to the high feed digestion. However, further studies are required to enhance our understanding of the bacterial functions and its relation to dietary nutrients.

In conclusion, the present study confirmed the hypothesis that the bacteria community and fermentation products *in vitro* could be altered by feeding isocaloric diets that differed in glucogenic and lipogenic nutrient content. When the glucogenic nutrient was above 1/3 of the energy source, the best feed digestion traits, as well as a lower acetate to propionate ratio, were obtained. The amylolytic bacteria including *Selenomonas*, *Succinivibrio* and *Ruminobacter*, as well as some cellulolytic bacteria including genera within the family *Ruminococcaceae*, the *Christensenellaceae_R‐7_group*, the *Eubacterium* and *some unclassified taxa* were more sensitive to the ratio of glucogenic to lipogenic nutrients.

## Conflict of Interest

The authors have no conflicts of interest.

## Author contributions

Conceptualization, D.H.; methodology, D.H.; data collection: D.H., Y.W., F.X. and Y.Z.; writing, review and editing, D.H., Y.Z. and X.N.; supervision, L.J. and B.X.; project administration, L.J. and B.X.; All authors have read and agreed to the published version of the manuscript.

## Nucleotide sequence accession number

All raw sequence files were submitted to the NCBI (National Centre of Biotechnology Information) Sequence Read Archive (SRA) database (Accession number, PRJNA661445).

## Supporting information

Figure S1 The *in vitro* gas production machine with automated gas production recording system.
Table S1 Effect of glucogenic to lipogenic nutrient ratios on the relative abundances of bacterial phyla in rumen fluid (%).
Table S2 Effects of different glucogenic to lipogenic nutrient ratios on the relative abundance of the KEGG* pathways of ruminal bacteria.
