# Supplementary material for: Effect of different glucogenic to lipogenic nutrient ratios on rumen fermentation and bacterial community in vitro
Source: J Appl Microbiol. 2020 Nov 21;130(6):1868–82. doi: 10.1111/jam.14873 (PMC8247007; doi:10.1111/jam.14873)
Supplement: Supplementary file 1 — Figure S1 The in vitro gas production machine with automated gas production recording system. Table S1 Effect of glucogenic to lipogenic nutrient ratios on the relative abundances of bacterial phyla in rumen fluid (%). Table S2 Effects of different glucogenic to lipogenic nutrient ratios on the relative abundance of the KEGG* pathways of ruminal bacteria. [file JAM-130-1868-s001.docx]

Effect of different glucogenic to lipogenic nutrient ratios on rumen fermentation and bacterial community *in vitro*

**Figure S1a,S1b.**

The *in vitro* gas production machine with Automated Gas Production Recording System.

**Figure S1a**


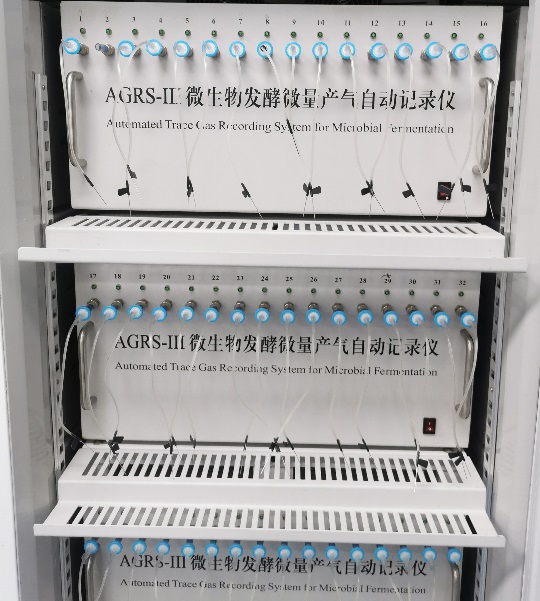


**Figure S1b**


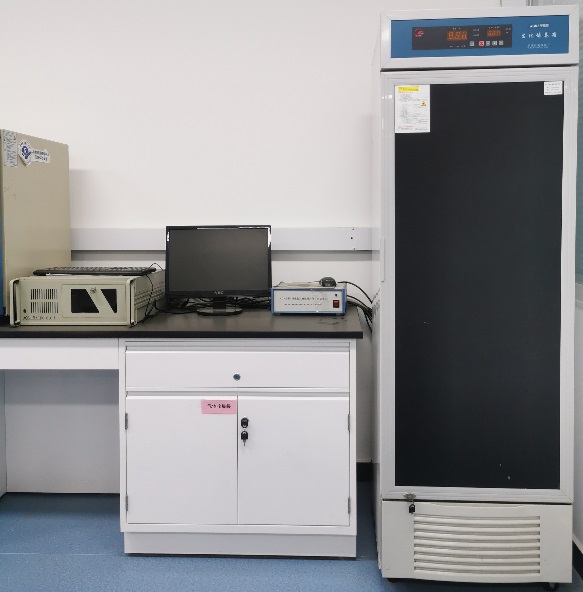


**Table S1**

Effect of glucogenic to lipogenic nutrient ratios on the relative abundances of bacterial phyla in rumen fluid (%)

| **Phyla** | **Relative abundance %** | | | | **SEM** | ***p-*value** |
| --- | --- | --- | --- | --- | --- | --- |
|  | **G** | **GL1** | **GL2** | **L** |  |  |
| *Bacteroidetes* | 50.88 | 47.37 | 45.05 | 44.48 | 0.948 | 0.089 |
| *Firmicutes* | 33.19^b^ | 39.19^ab^ | 42.12^a^ | 42.27^a^ | 1.272 | 0.039 |
| *Kiritimatiellaeota* | 3.99 | 3.68 | 3.59 | 3.27 | 0.302 | 0.904 |
| *Proteobacteria* | 4.72^a^ | 2.95^b^ | 2.31^bc^ | 1.32^c^ | 0.333 | 0.001 |
| *Epsilonbacteraeota* | 2.34 | 2.75 | 2.27 | 3.88 | 0.620 | 0.910 |
| *Spirochaetes* | 2.42 | 1.15 | 1.57 | 1.64 | 0.233 | 0.392 |
| *Patescibacteria* | 1.06^b^ | 0.92^b^ | 0.93^b^ | 1.46^a^ | 0.066 | 0.010 |
| *Synergistetes* | 0.32 | 0.43 | 0.50 | 0.54 | 0.048 | 0.314 |
| *Lentisphaerae* | 0.42 | 0.33 | 0.46 | 0.48 | 0.035 | 0.299 |
| *Actinobacteria* | 0.13^b^ | 0.61^a^ | 0.69^a^ | 0.14^b^ | 0.073 | 0.002 |
| *Tenericutes* | 0.29^b^ | 0.27^b^ | 0.32^b^ | 0.5^a^ | 0.028 | 0.094 |
| *unclassified_k__norank_d__Bacteria* | 0.05^b^ | 0.14^ab^ | 0.17^a^ | 0.05^b^ | 0.020 | 0.088 |
| *Elusimicrobia* | 0.06 | 0.04 | 0.09 | 0.09 | 0.009 | 0.101 |
| *Cyanobacteria* | 0.05 | 0.09 | 0.06 | 0.05 | 0.007 | 0.440 |
| *Chloroflexi* | 0.02^b^ | 0.08^ab^ | 0.10^a^ | 0.02^b^ | 0.012 | 0.052 |
| *Fibrobacteres* | 0.06^a^ | 0.02^b^ | 0.02^b^ | 0.04^ab^ | 0.006 | 0.082 |
| *WPS-2* | 0.013^bc^ | 0.031^a^ | 0.019^b^ | 0.007^c^ | 0.002 | 0.000 |
| *Armatimonadetes* | 0.008 | 0.010 | 0.013 | 0.013 | 0.0017 | 0.757 |
| *Planctomycetes* | 0.002 | 0.002 | 0.001 | 0.003 | 0.0006 | 0.763 |
| *Verrucomicrobia* | 0.004 | 0.001 | 0.001 | 0.002 | 0.0007 | 0.465 |

G, glucogenic diet; GL1, glucogenic: lipogenic ingredient = 2: 1; GL2, glucogenic: lipogenic ingredient = 1: 2; L, lipogenic diet. ^a,b,c^ means values with different letters differed significantly within a row (*p* < 0.05); SEM = standard error of the mean.

**Table S2**

Effects of different glucogenic to lipogenic nutrient ratios on the relative abundance of the KEGG* pathways of ruminal bacteria

| **Metabolic pathways** | **Diets**** | | | | **SEM** | **p-value** |
| --- | --- | --- | --- | --- | --- | --- |
|  | **G** | **GL1** | **CL2** | **L** |  |  |
| Metabolism |  |  |  |  |  |  |
| Amino Acid Metabolism | 10.56 | 10.41 | 10.37 | 10.39 | 0.031 | 0.106 |
| Carbohydrate Metabolism | 9.98 | 10.00 | 9.99 | 10.00 | 0.030 | 0.997 |
| Energy Metabolism | 6.11 | 6.05 | 6.04 | 6.13 | 0.016 | 0.188 |
| Metabolism of Cofactors and Vitamins | 4.61^a^ | 4.5^b^ | 4.49^b^ | 4.53b | 0.016 | 0.011 |
| Nucleotide Metabolism | 4.38^a^ | 4.31^ab^ | 4.3^b^ | 4.29b | 0.012 | 0.016 |
| Glycan Biosynthesis and Metabolism | 3.03^a^ | 2.82^b^ | 2.76^b^ | 2.8b | 0.033 | 0.002 |
| Lipid Metabolism | 2.81 | 2.85 | 2.83 | 2.80 | 0.008 | 0.285 |
| Enzyme Families | 2.23 | 2.22 | 2.21 | 2.21 | 0.005 | 0.464 |
| Metabolism of Terpenoids and Polyketides | 1.80 | 1.77 | 1.76 | 1.76 | 0.006 | 0.228 |
| Metabolism of Other Amino Acids | 1.61^a^ | 1.55^b^ | 1.55^b^ | 1.57ab | 0.008 | 0.040 |
| Xenobiotics Biodegradation and Metabolism | 1.50^b^ | 1.56^a^ | 1.56^a^ | 1.49b | 0.009 | 0.000 |
| Biosynthesis of Other Secondary Metabolites | 1.05 | 1.03 | 1.02 | 1.02 | 0.005 | 0.071 |
| Genetic Information Processing |  |  |  |  |  |  |
| Replication and Repair | 9.41^b^ | 9.87^a^ | 10.07^a^ | 10.16^a^ | 0.024 | 0.005 |
| Translation | 6.4^a^ | 6.26^b^ | 6.24^b^ | 6.3b | 0.018 | 0.002 |
| Folding, Sorting and Degradation | 2.68^a^ | 2.62^ab^ | 2.6^b^ | 2.63^ab^ | 0.010 | 0.001 |
| Transcription | 2.33 | 2.47 | 2.49 | 2.41 | 0.020 | 0.007 |
| Environmental Information Processing |  |  |  |  |  |  |
| Membrane Transport | 9.72^a^ | 9.55^ab^ | 9.52^b^ | 9.51^b^ | 0.100 | 0.005 |
| Signal Transduction | 1.43^b^ | 1.5^ab^ | 1.52^a^ | 1.52^a^ | 0.011 | 0.014 |
| Signaling Molecules and Interaction | 0.16 | 0.16 | 0.16 | 0.15 | 0.001 | 0.370 |
| Unclassified |  |  |  |  |  |  |
| Cellular Processes and Signaling | 3.88^a^ | 3.82^b^ | 3.8^b^ | 3.81^b^ | 0.008 | 0.001 |
| Metabolism | 2.43^ab^ | 2.45^a^ | 2.45^ab^ | 2.4^b^ | 0.006 | 0.038 |
| Poorly Characterized | 4.87 | 4.83 | 4.83 | 4.80 | 0.012 | 0.371 |
| Genetic Information Processing | 2.69 | 2.69 | 2.69 | 2.68 | 0.002 | 0.724 |
| Cellular Processes |  |  |  |  |  |  |
| Cell Motility | 1.82^b^ | 2.23^a^ | 2.29^a^ | 2.20^a^ | 0.056 | 0.007 |
| Cell Growth and Death | 0.59^a^ | 0.57^b^ | 0.57^b^ | 0.58^ab^ | 0.003 | 0.024 |
| Transport and Catabolism | 0.40 | 0.38 | 0.37 | 0.37 | 0.005 | 0.180 |
| Organismal Systems |  |  |  |  |  |  |
| Endocrine System | 0.32 | 0.32 | 0.32 | 0.30 | 0.003 | 0.358 |
| Environmental Adaptation | 0.14 | 0.15 | 0.15 | 0.15 | 0.001 | 0.065 |
| Nervous System | 0.10 | 0.10 | 0.10 | 0.10 | 0.001 | 0.074 |
| Immune System | 0.09 | 0.10 | 0.09 | 0.09 | 0.001 | 0.352 |
| Digestive System | 0.05 | 0.05 | 0.05 | 0.04 | 0.001 | 0.164 |
| Excretory System | 0.04 | 0.04 | 0.04 | 0.03 | 0.001 | 0.583 |
| Circulatory System | 0.00 | 0.01 | 0.00 | 0.01 | 0.001 | 0.916 |

*KEGG = Kyoto Encyclopedia of Genes and Genomes.

**G, glucogenic diet; GL1, glucogenic / lipogenic ingredient = 2: 1; GL2, glucogenic / lipogenic ingredient = 1: 2; L, lipogenic diet.

^a, b^ Mean values within a row with dissimilar superscript lower-case letters are significantly different (P ≤ 0.05) in the analysis that comprised all the groups.
